# Transactional Sex Risk across a Typology of Rural and Urban Female Sex Workers in Indonesia: A Mixed Methods Study

**DOI:** 10.1371/journal.pone.0052858

**Published:** 2012-12-28

**Authors:** Dewi Ismajani Puradiredja, Ernestina Coast

**Affiliations:** 1 Department of Population Health, London School of Hygiene and Tropical Medicine, London, United Kingdom; 2 Department of Social Policy, London School of Economics and Political Science, London, United Kingdom; Indiana University and Moi University, United States of America

## Abstract

Context-specific typologies of female sex workers (FSWs) are essential for the design of HIV intervention programming. This study develops a novel FSW typology for the analysis of transactional sex risk in rural and urban settings in Indonesia. Mixed methods include a survey of rural and urban FSWs (n = 310), in-depth interviews (n = 11), key informant interviews (n = 5) and ethnographic assessments. Thematic analysis categorises FSWs into 5 distinct groups based on geographical location of their sex work settings, place of solicitation, and whether sex work is their primary occupation. Multiple regression analysis shows that the likelihood of consistent condom use was higher among urban venue-based FSWs for whom sex work is not the only source of income than for any of the other rural and urban FSW groups. This effect was explained by the significantly lower likelihood of consistent condom use by rural venue-based FSWs (adjusted OR: 0.34 95% CI 0.13–0.90, p = 0.029). The FSW typology and differences in organisational features and social dynamics are more closely related to the risk of unprotected transactional sex, than levels of condom awareness and availability. Interventions need context-specific strategies to reach the different FSWs identified by this study's typology.

## Introduction

Indonesia has one of the fastest growing HIV epidemics in Asia, and sexual transmission is among the primary modes of transmission [1]–[3]. Projections suggest that 43% of new HIV infections due to sexual transmission will be attributable to unprotected transactional sex by female sex workers (FSWs) and their clients by 2014 in Indonesia [3]. In some settings, up to 16% of Indonesian FSWs are infected with HIV, and more than 60% of FSWs do not consistently use condoms with clients [4], [5]. The importance of unprotected transactional sex in countries with concentrated HIV epidemics requires an improved understanding of the context and organisation of at-risk groups, such as female sex workers. Sex work typologies or distinctions between types of sex workers are usually taken into account as part of a study's sampling strategy [4], [6]–[9], but can also be considered as an independent variable in subsequent analysis on sex work, HIV/STI risk and associated behaviours [6].

HIV surveillance in Indonesia dichotomises female sex work into “direct” (primary occupation) and “indirect” (sex work is not the sole or primary source of income), although both are found in diverse settings. FSWs working in rural areas are currently excluded from the Indonesian typology, as national surveillance samples only from urban sites [4], [5], [8]. Yet FSWs are likely to have different behavioural patterns in different contexts. The setting in which FSWs engage clients may affect FSWs' ability to negotiate condom use, as well as client numbers and type [6], [9], [10].

As elsewhere in Asia [11], [12], rural sex work exists in Indonesia, where more than half the population is rural [13] and where there is considerable circular migration, including by FSWs and their clients [5], [14]. Therefore, there is an urgent need to extend HIV risk behavioural research in Indonesia to include rural FSWs.

This study builds on the literature addressing sex work typologies [6], [8], [9], and aims to 1) examine differences in behavioural risk across a more differentiated typology of FSWs working in rural and urban sex work settings; and 2), investigate social and structural determinants that explain these differences.

## Methods

### Ethics statement

Ethical approval was granted by the London School of Economics (LSE) Research Ethics Committee on 04 August 2006 and verbal informed consent obtained from all participants.

### Data and sample

The study was conducted in two sites in West Java, Indonesia, selected to represent one urban and one rural context. Site selection was based on secondary analyses of Indonesian Behavioural Surveillance Survey (BSS) data [15], [16] and in consultation with the Centre for Health Research at University of Indonesia (CHR-UI) and local AIDS non-governmental organisations (NGOs).

The sample of FSWs was drawn up using a combination of purposive sampling techniques. First, ethnographic mapping identified locations where different types of FSWs solicit. Access to FSWs was established with the assistance of NGO outreach workers who already had trust relationships with FSWs and their ‘gatekeepers’, such as *preman* (hoodlums/thugs), managers or *germo* (pimps). The second stage of sampling, purposively selected sex work locations during pre-defined time intervals and then randomly selected survey respondents (n = 310) from each time-location unit for the quantitative survey, based on the principles of Targeted Sampling and Time-Location-Sampling (TLS) [17]. The third and final stage of the sampling process involved the stratified purposeful selection of a qualitative sub-sample of FSWs (n = 11) [18]. To this end, the survey sample was stratified by selecting ‘information rich’ cases according to different types of sex work setting who would illustrate the different FSW sub-groups and facilitate comparisons. A sampling grid was kept, in which each type of sex work setting represented one stratum. To the extent possible, the number of respondents per stratum was determined by the degree of theoretical saturation – that is, the degree to which new conceptual insights were generated per each additional respondent [19]. The response rate was 100% in both sites for both survey and in-depth interviews. Key informant interviews (n = 5) were conducted with former FSWs, NGO staff and health care providers.

Survey data were collected by the first author and two fieldworkers (one female, one male) from rural and urban FSWs (n = 310) between August and November 2007. Concurrently, in-depth interviews were conducted in the *Bahasa Indonesia* language using a semi-structured interview guide. Interviews were recorded, transcribed and translated into English. Data from interviews were accompanied by observational data, including field notes collected during repeat visits to both urban and rural sex work sites.

### Analyses

Qualitative data were analysed using a constant comparative thematic approach to define theoretical categories [20], [21]. Transcripts and field notes were read line-by-line and coded to delineate properties of different sex work contexts in relation to condom use. Data were open-coded into categories (e.g.: organisational structure of sex workplaces, inter-personal relationships at sex workplaces) which were repeatedly compared across the different sex work settings. Anonymised quotes from interview transcripts are presented in the results, and representative quotes have been selected from a variety of respondents in order to avoid selectivity in the range of data presented. Observations recorded in field notes are reported, and identified by (*sex work location, field note page number, date)*, for example (*Rural venue-based FSW, pp. 13–14, 02/11/07*).

Analysis of survey data was conducted using SPSS v. 19.0. Descriptive statistics were produced to compare individual-level socio-demographic characteristics, and establish variations in HIV behavioural risk, such as condom use and client volume. Chi-square tests were used for the categorical data, Mann-Whitney-U tests for the continuous data, and Fischer's Exact for sample counts of less than 5. For all analyses a *p*-value of <0.05 was taken to be statistically significant.

Multiple regression examined associations between consistent condom use in different settings, controlling for occupation-related and socio-demographic background characteristics; and, condom awareness, availability, and willingness to use. Our regression analysis sample was limited to respondents who reported to know what a male condom is (n = 305, 98% of survey sample). Since condom use will only offer reliable protection against HIV infection if used consistently, condom use frequency was dichotomised to distinguish those who reported never, sometimes and often having used condoms, from those who had always used condoms with their clients. Non-consistent condom use was coded as 1. Consistent condom use was coded as 0. Associations of consistent condom use with other variables were estimated using odds ratios with 95% confidence intervals (CIs) in logistic regressions. Firstly, univariate models containing only one explanatory variable were used to investigate the effect each factor alone had on the response: (non-) consistent condom use. Secondly, a multiple regression model was developed to determine effects of independent factors on (non-)consistent condom use controlling for other factors in the model. All the explanatory variables were included into the model on conceptual grounds. The final multiple logistic regression analysis included 9 explanatory factors to investigate the role of types of FSW settings after accounting for the influence of behavioural, socio-demographic and occupational-related background factors in predicting the likelihood of consistent condom use.

In keeping with our mixed methods approach, our analyses first describe urban and rural FSWs and their settings, and the implications for condom use, drawing on both quantitative and qualitative data. Then, we analyse the determinants of consistent condom use.

## Results

### Study population characteristics

Urban FSW are, on average, younger than their rural counterparts, and better educated (Table 1). Most urban FSWs are inter-provincial migrants (84%) whereas the majority of rural FSWs are local non-migrants (73%). The majority of both urban and rural respondents are divorced. The overwhelming majority of the study population is Muslim, and solicit from a range of places.

**Table 1:** Study population characteristics, stratified by urban and rural FSW populations (*n* = 310), 2007.

	Urban		Rural		*P* (a)
	n	%	n	%	
**Total**	184	59.4	126	40.6	-
**Age (years)** (b)	22	(19–29)	26	(22–30)	**<0.001**
**Place of solicitation**					
Brothel	33	17.9	0	0	**<0.001**
Street/rail tracks/rice fields	50	27.2	11	8.7	
Beauty/massage parlour/spa	68	37.0	0	0	
Karaoke/bar/cafe/street stall	33	17.9	112	88.9	
‘Freelance’	0	0	3	2.4	
**Educational attainment**					
No schooling/below primary	23	12.5	55	43.7	**<0.001**
Completed primary school	69	37.7	58	46	
Completed junior high	56	30.6	11	8.7	
Completed high school	35	19.1	2	1.6	
**Marital status**					**<0.001**
Married	30	16.3	3	2.4	
Divorced	79	42.9	104	82.5	
Widowed	6	3.3	4	3.2	
Separated	2	1.1	5	4.0	
Never married	67	36.4	10	7.9	
Multiple divorce (>1)	9	7.8	38	32.8	
**Migration status**					**<0.001**
Local	29	15.8	92	73.0	
Intra-province	0	0	18	14.3	
Inter-province	155	84.2	16	12.7	
**Religion**					
Muslim	181	98.4	126	100	**0.274**
Christian (Protestant)	3	1.6	0	0	

Notes:

(a) *P* values obtained from Chi-square and Mann-Whitney U test.

(b) Median age and inter-quartile range.

### Constructing a revised FSW typology

To develop a typology of Indonesian FSWs that moves beyond the direct/indirect dichotomy both geographical location (rural/urban) and whether place of solicitation is venue-based (VB) or non-venue-based (non-VB) should be considered. Sex-work venues include brothels, karaoke bars and spas, whilst non-venue based locations include rail tracks and rice fields. We identified five distinct sub-groups: (1) rural direct venue-based, (2) rural direct non-venue-based, (3) urban indirect venue-based, (4) urban direct venue-based, and (5) urban direct non-venue-based FSWs. If we apply this more detailed typology to our data, we see significant risk behavioural, socio-demographic and structural differences between FSWs in both quantitative and qualitative data. Table 2 summarises key characteristics of our proposed typology.

**Table 2:** Typology of Rural and Urban FSWs

Female sex work typology	1	2	3	4	5
**Geographical location**	**Rural**	**Rural**	**Urban**	**Urban**	Urban
**Mode of operation**	**Direct**	**Direct**	**Indirect**	**Direct**	Direct
**Place of solicitation**	**Venue-based:**- Music cafes- Warung	**Non-venue-based:**- Outdoors(e.g. in the rice fields)	**Venue-based:**- Spa- Massage parlour- Karaoke/Bar	**Venue-based:**- Brothels	Non-venue-based:- Street- Rail tracks
**Place of sex**	Indoors	Outdoors	Indoors	Indoors	Indoors/outdoors
**Site accessibility** (a) **(e.g. to research, outreach)**	High effort	High effort	Low effort	Medium to high effort	High effort
**Organisational structure**	- Semi-regulated,e.g. FSWs are accountable to pimp or venue-owner but there are no formalised agreements (i.e. written contracts); ‘freelancing’ FSWs are only accountable to respective venue-owner in that they have to pay the rent for booking a bedroom- services available all day but busiest time is after night fall	- Not regulated, e.g. FSWs work independently or have informal agreements with local pimps and hoodlums- work hours start from night fall (6 pm)	- Regulated,e.g. set working hours- set payment schemes- FSWs have to reach ‘quotas’- controlled client-FSW relationships- busiest work hours are from late afternoon until late	-Strongly regulated, e.g. strict working hours; FSWs live and eat on site (pay deducted from FSWs' earnings); savings are kept with managers; controlled client-FSW relationships- work hours start after night fall	- Not regulated, e.g. informal agreements with pimps; FSWs are (relatively) free to turn up for work or not- work hours start with nightfall (ca. 6 pm)
**Autonomy from employer** (b)	Medium	High	Medium to high	Low	High
**Personal safety** (c)	Medium	Medium	Medium (on-site);Low (off-site)	Medium	Low
**Interference from law enforcement** (d)	Medium	Medium	Low	Medium	High
**Type of clients**	Local fishermen and farmers; transient truck drivers	Local fishermen and farmers;transient truck drivers	Local and foreign businessmen; military, police and government staff	Local clients of lowersocio-economic status	Local clients of lowersocio-economic status
**Pay per transaction** (f)	IDR 50–100 000 (USD 5–10)	IDR 15–20 000 (USD 1.6–2)	IDR 250 000+(USD 27)	IDR 100 000(e) (USD 10)	IDR 30–70 000 (USD3–7.5)
**STI/HIV service coverage (incl. outreach)**	- Lack of STI/HIV services (incl. AIDS NGO outreach)	- Lack of STI/HIV services(incl. AIDS NGO outreach)	- Some sites have peer educators and/or offer regular health check-ups e.g. by private doctors or mobile NGO clinics with HIV/STI testing facilities	- Little HIV educational prevention facilities- Few sites have peer educators and/or offer regular health check-ups e.g. by private doctors, mobile clinics with HIV/STI testing facilities	- Lack of STI/HIV services(incl. AIDS NGO outreach)
**Condom access**	- Supermarkets and pharmacies- Not within easy walking distance	- Supermarket and pharmacies- Not within easy walking distance	- Available for purchase on-site and within easy walking distance	- Available for purchase at street stalls on site or nearby	- depending on location available from street stalls and supermarkets- distance varies

(a) As expressed by the effort it takes outreach workers and researchers to gain access to sites.

(b) Definition of autonomy: freedom of FSW to determine their own actions and behaviours; *High* autonomy = e.g. FSW can reject clients; can come to work as she likes (needs to); can leave the workplace after work hours; *medium* autonomy = e.g. FSW can reject clients and can leave the workplace after work hours but is otherwise pressured, i.e. through quota and payment schemes; *low* autonomy = e.g. FSW can only reject non-paying clients; cannot leave the workplace as she likes; are not free to decide on what to spent their earnings.

(c) Definition of personal safety: *High* e.g. can seek protection from police, manager, venue-owner, thugs and/or peers; *medium* e.g. can seek protection from venue-owner, pimp, thugs and/or peers; protection from police interference (i.e. raids); *low* e.g. cannot seek protection and subject to police harassment (i.e. raids).

(d) Definition of interference from law enforcement: *High* = frequent police raids and arrests, *Medium* = regular collection of bribes, occasional police raids, *Low* = occasional bribes, police raids uncommon.

(e) Based on the highest proportion of reported pay by last client in 2007. IDR = Indonesian Rupiah. USD = US Dollar.

(f) This is the gross price per transaction among direct venue-based urban FSWs. After compulsory deductions from the part of their employer, the net amount an FSW is to keep can be as little as IDR 10 000 per transaction.

#### Rural direct venue-based female sex work

In the rural site, FSWs most commonly work in venue-based settings, such as music cafes or *warung remang* (“shady” street stalls selling food or small goods) along a main highway, but also inland in more remote areas or in fishing villages along the coast. They are typically accountable to a *germo* (male pimp), *mamih* (female pimp), manager or café owner. In addition, some FSWs work as “freelancers,” who do not have a manager, but solicit clients independently in venues such as bars, music cafes and *warung remang*, and rent rooms in these sites, go to clients' homes or hotels, or work outdoors. Such “freelancers” can be local but are also FSWs who temporarily move from the cities to the countryside during the Muslim fasting month of Ramadan, when it is more difficult to work in cities due to increased police raids. Freelancing FSWs are generally welcomed by venue owners, as they attract clients and pay rent for bedrooms. The freelancing FSWs make informal agreements with local managers, and experience greater autonomy than FSWs on site, who have to adhere to specific workplace rules regarding work hours and payment.

The level of interference from law enforcement varies. Police raids can occur, particularly among the more accessible sites along the highway, but more commonly venue owners have informal agreements with the local police, involving small regularly collected bribes.

Venue-based FSWs tend to work in bedrooms adjacent to the cafes' entertainment areas. Sexual services are available all day but the busiest working hours tend to be after sunset, when their typical client base - local farmers, fishermen, and transient truck drivers – finish their work for the day. Although this study did not collect data from FSW clients, it became clear from rural peer educators and health care providers that prevention efforts addressed at the general population and male clients in particular were tentative at best, and levels of HIV/STI awareness and condom use acceptability low.

“It's difficult to make them (the clients) aware. If we were to go to the villages to try and raise awareness about condoms by telling the men straight out “you must use condoms” – that'd be unwise. I simply work on site. If they (the clients) come and want to get a room, without much chit-chat, I'll hand him a condom. He might ask “What's that for?” and I'd say “Prevention! Just use it!” – I just say that. I don't know whether they then actually use it or not. If I talk too much, I'm afraid they might argue, ‘cause the clients around here are just simple people – farmers and fishermen.”

- Rural peer educator, p. 12, 30/10/07

Condoms can be bought at local supermarkets and pharmacies, however, these are often not within easy walking distance, in particular in the more remote sites. A fifth (21%) of rural FSWs reported their main source of condoms as a local AIDS NGO, but the limited capacity of the NGO prevents them from providing adequate supplies of condoms (*Rural venue-based FSW, p. 15, 02/11/07*).

#### Rural direct non-venue-based female sex work

While the wider structural conditions, such as the lack of sexual health care provision and the degree of law enforcement, also apply to the rural non-venue-based FSWs, we observe marked differences in work organisational features and socio-economic positioning. Rural non-venue based FSWs solicit clients outdoors, e.g. in rice paddies. Non-venue-based FSWs solicit clients, for example, by sitting along a dirt path in the dark between the paddy fields, and clients step out a few meters into fields to have sex. Interaction time is minimal due to the relative discomfort of the location, thus negatively affecting willingness to suggest condom use, as this would prolong the transaction.

While rural non-venue-based FSWs can have informal agreements with local pimps, this type of sex work is less regulated and it is possible for these women to work more autonomously as compared with their rural venue-based peers. However, the amount of pay per client is on average much lower among the rural non-venue-based respondents than among their venue-based peers (Table 2). Incomes are affected by seasonality, especially during Ramadan and the rainy season, when the number of clients served outdoors drop considerably. Considering that a pack of condoms in local supermarkets and pharmacies can cost almost as much (IDR 6000–13 500/USD 0.6–1.35) as what a non-venue-based rural FSW would earn per transaction (IDR 15 000/USD 1.5) rural FSWs typically do not have enough condoms immediately at their disposal.

#### Urban indirect venue-based female sex work

Sex work in urban settings is more diverse than in rural areas (Table 2). The indirect FSWs included in this study work in spas, massage parlours, karaoke bars and bars, and can often obtain room and board from the owners of the venue in exchange for a percentage of their salary. Sexual transactions provided on-site take place in the bedrooms of these establishments. The clients of this group of FSWs included mostly local and foreign businessmen, but also government officials, military and police staff.

Among urban indirect venue-based FSWs in massage parlours and spas, sexual transactions are preceded by a massage or treatment, before the client can request ‘*plus-plus*’ services, which can range from non-penetrative (i.e. hand job) to penetrative sex (i.e. oral, vaginal, and anal). Venues usually have “menus” with photographs of their FSW employees, from which clients make their selection. Payments can be made using cash or credit card, with little opportunity for clients to abscond without payment. The rooms provide relative comfort and good lighting. Interaction times tend to be longer than in non-venue based sex work, providing more favourable preconditions for successful negotiation of condom use. Most of the indirect FSWs interviewed have a supply of condoms in their lockers and tend to carry condoms with them. Upon request, condoms can also be provided by “room service”, if so requested by the clients. However, condoms provided through “room service” are not free of charge and are paid for by the FSW not the client (*Urban indirect FSW, p. 23, 23/10/07*). Indirect sex work sites appear more accessible to AIDS NGO outreach, and several of the sites included in this study had on-site peer educators (PEs). The sex work context of indirect FSWs is characterised by far greater personal safety than that of urban non-venue-based FSWs, who are more likely to be subject to police harassment. Owners of entertainment venues try to circumvent police interference by posing as legal establishments and through paying bribes. Furthermore, a considerable number of clients are high-profile, often with military or police backgrounds. Abuse and harassment are more likely to occur from within the establishment, for example, in the form of sexual favours demanded by male management:

“There are no special requirements needed (to become an indirect FSW). You just come by if there is a vacancy for a masseuse. Just like that. They will test whether the applicant can massage or not and sometimes the boss asks to have sex with the applicant (…). When (my former boss) was in charge, he always asked the new girls to “play” (have sex with him). (Once) a girl refused (…) (My former boss) fired her.

- Indirect urban FSW, p. 9, 21/09/07

Overnight sexual services, usually taking place in hotels or clients' homes, are also provided through private arrangements between FSW and clients. Although the FSW can keep all of the pay, her personal risk is considerably higher, as she cannot seek help from management or peers. One respondent told us about an incident where a client in a hotel attempted to drug her in order to video-tape sex without her consent (*Indirect FSW, pp. 8–9, 05/09/07*).

#### Urban direct venue-based female sex work

The organisation of urban direct venue-based female sex work is similar to indirect venue-based, in that the women work for a *germo* and live on site. Here too, condoms are available for purchase on site or nearby. However, the clients of this group tend to be local Indonesians of lower socio-economic status than those served in indirect settings. Further, the degree of autonomy an FSW in these work settings experiences is far lower. At these venues debt bondage is common as is coercive entry into sex work:

“Back (home) I was sold by a neighbour. He said I would be working in a shop or as a house maid, but really he was selling me to a pimp. At the time I was a virgin. (…) He promised me a big salary. I was tempted. When I arrived in Jakarta they brought me to a place with many girls. I thought ‘why do they bring me to such a place’. I wanted to go home, but I couldn't escape because there were so many guards. They put make-up on me and brought me to a hotel…Then I realised…I cried at first but after a while…well, you just accept it”

- Urban direct VB, pp. 7–8, 22/09/07

Furthermore, while women working at indirect sites are free to leave outside of work hours, women working at brothel-complexes are usually confined to their work sites. Indonesian criminal law is ambiguous with respect to venue-based sex work. Some brothel-complexes are semi-regulated and have police presence to ensure that no music is played on Friday evenings and that the Muslim weekly holy day is honoured. Access to these sites is easier for NGOs and national HIV surveillance compared with illegal sites. A female pimp who used to work as an FSW describes an illegal brothel complex as follows:

“In [brothel complex X.] the kids (young FSWs) are wilder. […] It is very difficult to get (access) into X., very difficult to provide information on HIV/AIDS in there. It's a mix of people who run that place. They are thugs from (different parts of Indonesia). All sorts of people. They have complete control of the place. They even fight against the police. Everyone is scared of them. […] Also the NGOs.”

- Female pimp/Former urban direct VB FSW, p 5, 23/08/07

#### Urban direct non-venue-based sex work

In the urban site, non-venue-based FSWs included in this study, solicit clients on the street or along rail tracks. Street-based FSWs are considerably younger than those working along rail tracks. Clients of street-based FSWs can be Western or wealthier Indonesians, resulting in better pay than for rail-based sex workers, who tend to attract clients of lower socio-economic status. Non-venue based work is less formalised than venue-based. Women may be associated with a *germo* or *preman* (hoodlum, thug) to be allowed to work in the area. Often the FSWs form smaller groups under the ‘guardianship’ of a more senior (more experienced and older) FSW. Clients solicited on the street are typically served in nearby hotels or clients' homes while FSWs working along the rail tracks have sex with clients in small shacks adjacent to the tracks. There is no lighting, which may affect whether a condom is used correctly. One of our respondents mentioned how she would need to hold on to the condom while manoeuvring with a client in the shack so it ‘doesn't get lost in the dark’ (*Urban direct non-venue based, p. 31, 21/08/07*). Since soliciting clients in public places is illegal in Indonesia, non-venue-based sex work sites are frequently subject to police raids. Women working at the non-venue-based sites, whilst comparatively autonomous, experience abuse, harassment and violence from which they cannot seek police protection for fear of being arrested. This accentuates power imbalances between FSW and others in the work site, jeopardising negotiation of safe sex:

“Girls around here often don't get paid. A lot of the *preman* are like that…The girls complain that they've been ‘used’ by them without getting paid.”

- Direct non-VB FSW, p. 12, 21/08/07

While condoms can be purchased at nearby *warung* (street stalls) or supermarkets, women involved in urban non-venue based sex work tend only to carry condoms with them when working if they had been given them by NGO outreach workers. However, non-venue based FSWs are rarely covered by NGO outreach. At the few sites with NGO outreach presence, we observed as little as one or two NGO outreach workers attempting to reach hundreds of FSWs with condom distribution and HIV/AIDS educational information and material.

### Correlates of consistent condom use

Of the 305 (98.4%) respondents included in the formal statistical analyses, just over three quarters (76.4%) of respondents report to not always using condoms with clients during the week prior to interview. The age distribution among those FSWs who use condoms consistently and those who do not is similar (median age = 24 years) (Table 3). The majority of all respondents are aware that condoms can prevent sexual HIV acquisition and report having condoms available when working. A higher proportion of FSWs who use condoms consistently report to have proposed condom use to their clients prior to the sexual transaction (90.3%), whereas FSWs who do not use condoms consistently are more likely to not propose condom use to clients (54.9%).

**Table 3:** Respondent characteristics and determinants of condom use, stratified by consistent and non-consistent condom users (*n* = 305), 2007.

	Consistent users		Non-consistent users		*P* (a)
	n	%	n	%	
**Total** (b)	72	23.6	233	76.4	**-**
**Age (years)** (c)	24	(20–29)	24	(20–29)	**0.276**
**Condom awareness**					**0.075**
Know can prevent HIV	57	79.2	159	68.2	
Don't know	15	20.8	74	31.8	
**Condom availability**					**0.075**
Yes (Best case)	62	86.1	153	65.7	
No (Best case)	10	13.9	80	34.3	
**Willingness to suggest condom use**					**<0.001**
Always proposed use to clients last wk	65	90.3	128	54.9	
Not always proposed use	7	9.7	105	45.1	
**Type of sex work setting**					**<0.001**
Urban direct venue-based	5	6.9	28	12.0	
Urban direct non-venue-based	11	15.3	38	16.3	
Urban indirect venue-based	44	61.1	57	24.5	
Rural direct venue-based	10	13.8	101	43.3	
Rural direct non-venue based	2	2.8	9	3.9	
**Monthly SW income (USD)** (d)					**<0.001**
<50	4	5.6	24	10.4	
50–150	15	21.1	97	42.2	
150–250	22	31.0	66	28.7	
>250	30	42.3	33	18.7	
**Number of clients per week**					**0.057**
Less than 5	36	50.7	84	36.0	
5–10	21	29.6	98	42.1	
>10	14	19.7	51	21.9	
**Marital status**					**<0.001**
Married	17	23.6	14	6.0	
Divorced	33	45.8	149	64.0	
Widowed	2	2.8	7	3.0	
Separated	3	4.2	3	1.3	
Never married	17	23.6	60	25.7	
**Educational background**					**<0.001**
No schooling	2	2.8	16	6.9	-
Below primary school	4	5.6	53	22.8	
Completed primary school	24	33.3	102	44.0	-
Completed junior high	24	33.3	43	18.5	-
Completed high school	18	25.0	18	7.8	
Above high school	-	-	-	-	

**Notes:**

(a) *P* values obtained from Chi-square, Mann-Whitney U and Fisher's Exact tests as appropriate.

(b) 5 missing values, of which 4 reported to not know what a male condom is, and 1 who reported to not remember.

(c) Median age and Interquartile Range (IQR).

(d) 4 missing values: 1 among the consistent users and 3 among the non-consistent users. 1 USD = ca. IDR 9, 560.00 on 6 Sept 2012 according to http://www.xe.com/ucc/convert/?Amount=1&From=USD&To=IDR Here converted from IDR*1000 to USD (rounded up to IDR 10 000 per 1 USD).

The majority of FSWs working in urban indirect sex work settings, such as beauty spas or massage parlours, use condoms consistently, while the highest proportion of FSWs working in rural direct venue-based settings, such as cafes or street stalls, do not use condoms consistently. While most of the FSWs who use condoms consistently have a monthly income of at least IDR 1 500 000 (USD 150), the majority of those who do not use condoms consistently, earn IDR 500 000–1 500 000 (USD 50–150) and less. Average client volume does not differ substantially if segregated by consistent condom use: the majority report fewer than 10 clients in the week prior to interview, though a higher proportion of FSWs who use condoms consistently have fewer than 5 clients a week compared to those FSWs who do not use condoms consistently (50.7% and 36.0% respectively). A higher proportion of currently married respondents are found among FSWs who use condoms consistently (23.6%) as compared to those who do not. Levels of educational attainment are lower among FSWs who do not use condoms consistently.

Multiple regression analysis shows that the likelihood of consistent condom use is higher among urban venue-based FSWs for whom sex work is not the only source of income than for any of the other rural and urban FSW groups. This effect is explained by the significantly lower likelihood of consistent condom use by rural venue-based FSWs (adjusted OR: 0.34 95% CI 0.13–0.90, *p* = 0.029), after adjustment (Table 4).

**Table 4:** Unadjusted and adjusted odds ratios (ORs) for consistent condom use with clients, 2007.

Measure		Unadjusted			Adjusted		
		OR	95% CI	*p*-value	OR	95% CI	*p*-value
**Age (categories)**	>34 years	1	-	-	-	-	-
	25–34 years	0.93	(0.38, 2.29)	0.880	1.23	(0.37, 4.10)	0.733
	14–24 years	0.97	(0.41, 2.31)	0.943	0.96	(0.28, 3.36)	0.954
**FSW typology** (a)	Urban indirect venue-based	1	-	-	-	-	-
	Urban direct venue-based	0.55	(0.20, 1.47)	0.232	0.48	(0.15, 1.54)	0.216
	Urban direct non-venue-based	0.94	(0.45, 1.95)	0.870	0.85	(0.30, 2.39)	0.761
	Rural direct venue-based	0.23	(0.11, 0.46)	<0.001	0.34	(0.13, 0.90)	0.029
**Marital status**	Never married	1	-	-	-	-	-
	Married	4.84	(2.25, 10.41)	<0.001	4.16	(1.33, 13.04)	0.014
	Divorced, separated or widowed(b)	0.52	(0.30, 0.89)	0.017	2.33	(0.92, 5.91)	0.075
**Education level**	Below primary school level	1	-	-	-	-	-
	Completed primary school	0.64	(0.37, 1.11)	0.111	2.09	(0.69, 6.39)	0.194
	Completed junior high school	2.20	(1.22, 3.97)	0.009	3.13	(0.97, 10.13)	0.057
	Completed senior high school	3.96	(1.93, 8.13)	<0.001	5.25	(1.44, 19.11)	0.012
**Monthly income (USD)** (c)	>250	1	-	-	-	-	-
	250 - 150	0.77	(0.43, 1.35)	0.358	0.55	(0.24, 1.24)	0.150
	150 -50	0.44	(0.23, 0.83)	0.011	0.52	(0.20, 1.36)	0.184
	<50	0.55	(0.16, 1.94)	0.355	0.36	(0.67, 1.94)	0.234
**Number of clients per week**	1–5	1	-	-	-	-	-
	5–10	0.60	(0.35, 1.03)	0.062	0.35	(0.16, 0.80)	0.013
	>10	0.99	(0.51, 1.94)	0.984	0.33	(0.12, 0.90)	0.031
**Condom awareness**	Yes vs. No	1.77	(0.94, 3.33)	0.077	0.62	(0.27, 1.43)	0.260
**Condom availability**	Yes vs. No	2.00	(1.17, 3.43)	0.012	1.34	(0.66, 2.74)	0.423
**Suggest condom use**	Yes vs. No	11.32	(4.98, 25.73)	<0.001	12.56	(4.62, 34.15)	<0.001

(a) The category ‘rural direct non-venue-based FSWs’ was excluded from this model due to its small sample size (n = 11). A model, which included this sub-group, resulted in large estimates signalling sparse-data bias within this categorical stratum, before and after adjustment (unadjusted OR: 1.9E^−9^ 95% CI 0 - ∞, p = 0.999; adjusted OR: 2.6E^−9^ 95% CI 0 - ∞, p = 0.999). This made it difficult to draw any definitive conclusions. Further, given the detail of the ethnography, there is reason to suspect that the rural non-venue-based FSW sample is not representative in statistical terms.

(b) The categories divorced/separated and widowed were aggregated due to their similarity in the direction of effect.

(c) 1 USD = ca. IDR 9, 560.00 on 6 Sept 2012 according to http://www.xe.com/ucc/convert/?Amount=1&From=USD&To=IDR. Here converted from IDR*1000 to USD (rounded up to IDR 10 000 per 1 USD).

While age and income are not significantly associated with consistent condom use, currently married FSWs are four times as likely to use condoms consistently, than those who have never been married (adjusted OR: 4.16; 95% CI: 1.33, 13.04, *p* = 0.014). Those respondents who have completed senior high school are five times as likely to consistently use condoms as compared with those respondents who had received no schooling or not completed primary school (OR: 5.25; 95% CI: 1.44, 19.11; *p* = 0.012). Further, there is a trend that with increased educational attainment the likelihood of consistent condom use increases, and it decreases with increased number of clients.

Even before adjusting for the other factors in the model, condom awareness was not significantly associated with consistent condom use (unadjusted OR: 1.77; 95% CI: 0.94, 3.33; *p* = 0.077 and adjusted OR: 0.62; 95% CI: 0.27, 1.43; *p* = 0.260). Only whether condom use was consistently suggested to clients during the week prior to interview was significantly associated with consistent condom use, before and after controlling for other factors in the model (unadjusted OR: 11.32; 95% CI: 4.98, 25.73; p<0.001 and adjusted OR: 12.56; 95% CI: 4.62, 34.15; p<0.001). Thus, those who suggested condom use to clients had more than twelve times the odds of consistently using a condom compared to those who did not, although the wide confidence interval suggests some uncertainty.

### Contextualising condom use

Qualitative data revealed how contextual factors can have a bearing on FSWs' condom use decision-making, particularly economic reasons. Among urban direct venue-based FSWs, economic pressure is usually exerted by employers. Debt bondage ‘*kas bon*’ is common, with an advance paid by the pimp to prospective FSW. In return for the *kas bon*, the FSW must work only at that pimp's premises. A young, recently arrived FSW is unlikely to have any money, thus relies on an advance. This is paid in instalments, with rent, food and other expenses deducted from her salary, which means that of the IDR 110 000 (USD 11) earned per transaction, a FSW effectively gets to keep just IDR 10 000 (USD 1). If she decides to end her work at the premises, she will not see the remainder of the *kas bon*:

“The neighbour who had sold me, said I was sold for IDR 2 000 000. But I didn't receive the money. The first month I worked I didn't receive any pay at all. In the month thereafter they told me my debt was settled but I would have to pay 50% of my earnings to the pimp. For example, if I made IDR 200 000, we would have to give the pimp IDR 100 000 (…)

- Urban direct venue-based, pp. 4, 9–10, 12, 14, 18 22/09/07

While indirect venue-based FSWs at some sites may receive a fixed – if small - monthly salary the amount of money they can earn on top of that is substantially higher if they provide penetrative sex (up to millions of IDR if the FSW is a virgin or if she agrees to overnight services off-site) and subsequent tips, as compared to regular massage and spa services (typically around USD 0.50–0.75 per session) only (*Indirect urban, p. 7, 21/09/07*; *Indirect urban, p. 7, 05/09/07*).

Competition is high at indirect sites – especially between younger and older FSWs - as clients can request a specific woman rather than being assigned to one. As a result, less popular women may go days without clients.

“My income varies because I have many competitors who are a lot younger than me. It is possible for me to have no customers for three days (…) It's sad to get no clients although I am never absent from work. It's all because there are so many younger employees.”

- Urban indirect FSW, p. 5, 05/09/07

In addition, clients are fewer at indirect sites than at direct sites, and some may choose to have only a massage. At the same time, indirect FSWs can have quotas of clients served per month in order to keep their jobs. Thus, some women always accept client demands to boost their income. As one respondent phrased it:

“Clients don't want to use condoms…If a girl wants to have a lot of clients, she must be willing not to use condoms, because if she services well, other clients will hear about it by word of mouth”

- Urban indirect FSW, p. 5, 21/09/07

This implies that if an FSW refuses to have unprotected sex with a client, she may lose him to another FSW.

Existing research suggests that although individuals might use condoms successfully with particular partners, their use with regular or steady partners may be suboptimal [22]–[25]. The qualitative data found that the relationship between condom use and the type of client is not always clear-cut. Most respondents felt it is easier to suggest condom use to new clients (*e.g. Resp. 310249, 21/11/07; 395255, 21/11/07, Notes*). One reason to not use condoms with regular clients was that regular clients tend to pay better than new or infrequent clients. Indeed, becoming a “wife” or “girlfriend” (commonly used euphemisms for becoming a ‘mistress’) to regular clients is considered the most desirable sex work, resulting in considerably higher pay and extra gifts:

“It's good to become a mistress because it means we get financially supported”

- Urban direct venue-based, p. 16, 22/09/07

Regular clients are often considered ‘safer’, as they are assumed to have fewer partners by restricting their (extramarital) sexual relationships to them (*e.g. Resp. 310251, 21/11/07, Notes*). It is, however, problematic to introduce condom use into transactions with established regular clients (*e.g. Resp. 310247, 21/11/07*).

On the other hand, it was also suggested that it would be easier to use condoms with regular clients, as they would ‘care more’ for the well-being of the FSW due to greater familiarity and emotional attachment. In addition, condom use with new clients was said to be not advisable, as it could be ‘bad for business’ to insist on condom use the first time around, as it may mean that clients would be disinclined to use their services again (*e.g. Resp. 310245, 310247, 21/11/07; 314299, 24/11/07 - Notes*).

FSWs also emphasised that clients differ considerably and women have to make quick character judgments on the basis of a client's looks and demeanour to decide whether to suggest using condoms. One respondent told us that if a client's face looked *marah* (*lit.*: angry, *here*: aggressive), she would refrain from insisting on condom use (*Resp. 313234, Notes, 20/11/07*).

Irrespective of type, clients play an important role in making safe sex happen. In-depth interviews with the urban indirect FSWs revealed that clients who are aware and concerned about STIs are more likely to use condoms (*e.g. Indirect urban, p. 5, 21/09/07*). From the interviews it also emerged that clients with foreign backgrounds (i.e. Western and East Asian) and higher socio-economic status (i.e. businessmen) tend to be more likely to be open to using condoms. By comparison, the lack of HIV/STI awareness and, as a result, health concern, is particularly pronounced among the local clients of rural FSWs who are mostly farmers and fishermen of lower socio-economic standing.

“Unless they (the clients) are aware and don't want to catch a disease, no one seems to really care about not infecting others if they already have it (HIV/STI).”

- Rural peer educator, p. 9, 30/10/07

On the other hand, it can be the client who “educates” FSWs about how to use condoms and why. As one respondent pointed out:

“I first learnt about condoms from a client. He brought one with him and I thought it was chewing gum…I didn't even know how to put it on back then…that client taught me how to do it”

- Rural direct VB, 23/11/07, p. 26

Qualitative data suggest that condom use decision-making is not only influenced by FSWs' clients but also by other key actors at their workplaces. Overall, the brothel-owners and pimps tend to be mostly indifferent as to whether their employees use condoms with their clients or not, as long as it does not affect their business.

Problems arise if venue management does not support employees and sides with clients in cases of complaint:

“The manager sides with the client. If a client complains, we are being reprimanded. I hate guests that talk nice/sweet inside (the bedroom) but outside (the bedroom) they complain. The manager will believe (side with) the client, so that he will come back” (…)

*[Interviewer: “What happens if the client really doesn't want to use condoms?”]*

“I have to serve him then. I'm afraid he would complain at reception (otherwise)”.

- Urban indirect FSW, pp. 16 & 26, 23/10/07

The situation can be particularly precarious if the client holds particular authority, for example, by being a government official or member of the military or police, who could potentially arrange to have the business closed down. This is especially relevant in indirect sex work settings who tend to cater to this type of client base.

Among indirect venue-based urban FSWs the supportiveness of the manager was particularly important. We noted that at sites where the manager was supportive of condom use, women felt more empowered to refuse clients who did not want to use condoms. Interestingly, those managers that we identify as pro condom use tended to be female. As one respondent noted:

“We are required to use condoms with our clients. There was a case when a client had paid for the full service (massage and sex) (…). But when he was asked to use a condom, he refused. He got angry and talked to the manager. Mrs X (the manager) told that client that condom usage was mandatory here (at this venue)”

- Indirect FSW, p. 12, 05/09/07

Involvement of members from vulnerable groups in prevention efforts is an increasingly popular approach. The underlying reason is that when knowledge is imparted by an “insider”, it is perceived as less patronising and hence more acceptable. However, during our field site visits and in conversation with PEs it emerged that even in the presence of peer educators in some of the indirect sex work sites, it is challenging to reach a consensus among peers on condom use, which might have the desired normative effect on individuals' behaviour. This is in part attributable to perceived peer pressure in the form of competing for clients (*Former indirect urban FSW, pp. 11–12, 2006*), but may also be due to fatigue caused by over-saturation in terms of HIV educational measures addressed at these women (*Indirect urban FSW and PE, p. 4, 21/09/07*).

From conversation with pimps, and reiterated in the interviews with our FSW respondents, it emerged that most pimps at the venue-based sites show a greater willingness to have doctors or mobile HIV/STI clinics attend their premises to have their employees checked up (all at the expense of their employees), than opening their venues up to NGO HIV/STI preventative outreach. Overall, there seems to be a greater motivation among pimps to check whether their ‘commodity’ (sex worker) is clean and available to generate revenue as opposed to having a genuine interest in preventing the spread of sexually transmitted diseases (*Female pimp, p. 1, 23/08/07*).

## Discussion

This paper proposes an FSW typology that extends understanding of FSW risk environments in Indonesia by including previously un-researched rural FSWs and by distinguishing more subtly between different FSWs and their work settings. FSWs in Indonesia are operating in various settings with heterogeneous socio-demographic backgrounds and differential risk behaviours. Most notably, our findings indicate that the likelihood of consistent condom-use varies by type of sex work setting: there is a significant difference in the likelihood of consistent condom use in urban indirect sex work settings as compared with the rural direct venue-based sex work settings.

This finding has a number of important implications. Firstly, disease surveillance systems and most intervention efforts in Indonesia primarily target urban venue-based FSWs. Rural FSWs and FSWs working in precarious non-venue-based urban settings are largely excluded from existing intervention efforts. These women are not as easily accessible as venue-based FSWs. Surveillance and intervention efforts will need to address the needs of these neglected sub-groups to improve HIV and STI mitigation.

Second, the results corroborate findings from previous research into the role of sex workplace – both in terms of its physical and structural particularities and the social meanings attached to it – in mediating FSWs' condom use behaviour [9], [10], [26]–[30]. The qualitative analysis suggests differences in social-structural profiles of rural and urban (non-) venue-based sex work settings, which may in part explain the different association. Self-contained environments, such as urban indirect establishment-based settings, are more accessible to AIDS NGO outreach programmes and are more likely to have condoms available for purchase than the rural direct sites. Indonesia's recent decentralisation of government services including health care, to the district level, has created inefficiencies in the rural health care system [31]. Local governments now define spending priorities. In the rural site, HIV and STI services are of relatively low priority, evidenced by the limited funding made available by the local government to address these issues, and the consequently low numbers of *puskesmas* (public health centre) and hospitals that offer basic STI/HIV-related health care services in rural areas. Hence, rural FSWs and the general rural population lack the basic prerequisites for safe sex, including convenient access to condoms.

While most sex work establishments can operate largely undisturbed, as long as the owners pay bribes to the local police, these police payments do not guarantee protection from campaigns, especially during Ramadan, when arrests are made more frequently, and extortionate bail demands are imposed by local law enforcement.

Indirect sex work sites tend to be less affected by restrictive law enforcement practices as the direct sex work settings, in particular the urban non-venue based sites, which are more frequently subject to police raids. Given that non-venue based work is officially prohibited in Indonesia, combined with activities of alcohol and drug selling and use common at these sites, these FSWs are a prime target for police arrests. Fear of police persecution can deter women from carrying condoms, as from our observations and elsewhere [31], condoms can be signifiers of prostitution. During one of our field trips we were caught in a police raid and falsely accused of being involved in prostitution on the basis of carrying large amounts of condoms. The more detrimental socio-structural working conditions among the urban direct sex work settings, for instance, due to police harassment, lack of managerial and social support, may help explain why these women might use condoms less consistently than those women who work at urban indirect sex work sites.

Further, while the results showed no significant association between the level of income and consistent condom use after adjustment (possibly in part attributable to methodological challenges in measuring income), findings are consistent with those seen in previous studies in that the likelihood of consistent condom use is significantly associated with levels of educational attainment and client volume. A number of studies have identified educational attainment as a strong socio-economic predictor of consistent condom use and other HIV-related outcomes [32]–[36]. Attending school raises health literacy, which is associated with lower-risk sexual behaviours, and may influence sexual networks that affect HIV risk [37]–[38]. Further, higher educated individuals can have greater access to economic resources and thereby decrease their dependence on relationships [39]. This is supported by our data, which show that FSWs working in urban indirect settings who have the highest educational attainment also have the highest income, are also more likely to use condoms consistently. It has also been well-established that number and composition of sexual partners are important determinant STI/HIV patterns [40]. This study's findings show that an increase in the number of clients decreases the likelihood of consistent condom use.

The association with marital status deserves further research. Married respondents were significantly more likely to use condoms consistently than those who had never been married. One simple explanation would be that respondents who are married are particularly careful not to have extra-marital pregnancies. However, this study's survey data as well as that of the Indonesian Demographic Health Survey [13], [41] suggest that condoms are unlikely to be used for contraceptive purposes. Extant research has cited marriage as having protective health effects, due to greater economic resources and social support and reputation [42], [43]. Thus, another possible explanation could be that single and divorced FSWs are more likely to engage in sex work so as to compensate for lost marriage income, which puts them in an economically disadvantageous position in relation to clients and their demands. Marital status has also been linked to HIV risk behaviour through mechanisms such as client volume. Results from previous research have shown that single FSWs were more likely to depend exclusively on sex work for their incomes and serve a greater number of clients than their married counterparts [44]. A greater client volume decreases the likelihood of consistent condom use, which was confirmed by higher numbers of single FSWs being HIV positive than among married ones [42], [43].

Finally, factors typically thought of as programmatically important, such as condom awareness and carrying condoms whilst working, were not significantly associated with consistent condom use before and after adjustment. The variable ‘willingness to suggest condom use’ was significantly associated, indicating the roles of individual agency and self-efficacy in condom use.

The qualitative findings showed how context-related factors shape decision-making. This comes as a result of complex interactions between individuals' socio-economic position and key actors within their work. During in-depth interviews it was telling how few comments were made by respondents' about HIV risk and its prevention through condom use, if not probed by the interviewer. By allowing respondents to express themselves more freely, factors were highlighted that FSWs perceived as important when negotiating their work environments. It emerged that the interviewees discussed condom use primarily in terms of material or economic concerns and the nature of interpersonal relationships. Future prevention efforts need to move beyond basic provision of condoms and educational campaigns targeted solely at FSWs, and also account for the socio-structural drivers that shape risk behaviour in transactional sex, including work organisational features, gatekeepers (managers, pimps and *preman*) support of condom use, the nature of interpersonal relationships (e.g. between FSWs and her peers and clients of varying socio-economic backgrounds), the socio-legal setting which discourages condom use for fear of police raids, and socio-economic disadvantage.

### Limitations

The current study has several limitations. The quantitative data are drawn from a cross-sectional survey, meaning that conclusions cannot be made regarding causality of relationships. Another limitation is that this study relied on self-reported sensitive behaviour, and responses are therefore susceptible to social desirability bias. Moreover, women were asked questions about events that occurred in the past, which may be difficult to recall. Further, the sample did not include clients, which prevented the opportunity to corroborate responses. However, different internal and external measures of validation were employed, to maximise the degree of validity and reliability of both the quantitative and qualitative findings of this study, including rapport-building with the study population through ethnographic field visits and collaborating with local outreach workers and key members of the study population, “triangulation” inherent in mixed methods research through the complementary use of both qualitative (in-depth interviews and ethnographic assessments) and quantitative (survey) methods, as well as external validation through triangulating survey results from this study for consistency with similar information on the overall spectrum of behaviour from secondary data sources, such as the Indonesian Behavioural Surveillance Survey (BSS) and Demographic Health Survey (DHS).

It is also important to note that, the study sample was not a population-based sample, since a sampling frame of all FSWs in the West of Java was not possible to construct due to the hidden nature of sex work. However, the purposive sampling design aimed to make the sample representative in terms of the study population's heterogeneous sex work contexts. Nevertheless, more research with samples in different rural settings outside West Java province in Indonesia would be useful to confirm findings. Further, while we attempted to sample ‘in depth cases’ with diverse backgrounds from each type of sex work setting, as well as a ‘peripheral’ sample of key informants who are not central to the phenomenon but associated with it, the final sample size was ultimately also determined by time and resources. For example, the qualitative sub-sample did not include in-depth interviews with FSWs in rural non-venue-based settings. This means that theoretical saturation of the qualitative sample was not necessarily achieved. However, consultation with NGO fieldwork assistants with extensive experience of the fieldwork setting suggested that the narratives of the women interviewed - and complemented by the key informant interviews - represented a wide range of FSW perspectives in the field sites.

## Conclusion

This study highlights the importance of a more differentiated typology of female sex work settings in predicting consistent condom use. It also has important programmatic implications for designing surveillance and intervention activities. FSWs working in rural direct venue-based sex work settings were identified as being at particular risk of unprotected sex, as compared with urban peers, due to organisational characteristics and social dynamics. Interventions need focused strategies across socio-ecological levels (e.g. individual and structural) to reach FSWs working in heterogeneous settings even in smaller geographical locations, including those working in rural areas.
